# Empiric antibiotic therapy in urinary tract infection in patients with risk factors for antibiotic resistance in a German emergency department

**DOI:** 10.1186/s12879-018-2960-9

**Published:** 2018-01-26

**Authors:** Sebastian Bischoff, Thomas Walter, Marlis Gerigk, Matthias Ebert, Roger Vogelmann

**Affiliations:** 1 Second Department of Internal Medicine, University Medical Center Mannheim, Medical Faculty Mannheim, Heidelberg University, Theodor-Kutzer Ufer 1-3, D-68167 Mannheim, Germany; 2 Emergency Department, University Medical Center Mannheim, Medical Faculty Mannheim, Heidelberg University, Theodor-Kutzer Ufer 1-3, D-68167 Mannheim, Germany; 3 Department of Microbiology, University Medical Center Mannheim, Medical Faculty Mannheim, Heidelberg University, Theodor-Kutzer Ufer 1-3, D-68167 Mannheim, Germany

**Keywords:** Antimicrobial resistance, Emergency medicine, Infectious disease, Multidrug resistance, Risk factors, Urinary tract infection, Antibiotic therapy

## Abstract

**Background:**

The aim of this study was to identify clinical risk factors for antimicrobial resistances and multidrug resistance (MDR) in urinary tract infections (UTI) in an emergency department in order to improve empirical therapy.

**Methods:**

UTI cases from an emergency department (ED) during January 2013 and June 2015 were analyzed. Differences between patients with and without resistances towards Ciprofloxacin, Piperacillin with Tazobactam (Pip/taz), Gentamicin, Cefuroxime, Cefpodoxime and Ceftazidime were analyzed with Fisher’s exact tests. Results were used to identify risk factors with logistic regression modelling. Susceptibility rates were analyzed in relation to risk factors.

**Results:**

One hundred thirty-seven of four hundred sixty-nine patients who met the criteria of UTI had a positive urine culture. An MDR pathogen was found in 36.5% of these. Overall susceptibility was less than 85% for standard antimicrobial agents. Logistic regression identified residence in nursing homes, male gender, hospitalization within the last 30 days, renal transplantation, antibiotic treatment within the last 30 days, indwelling urinary catheter and recurrent UTI as risk factors for MDR or any of these resistances. For patients with no risk factors Ciprofloxacin had 90%, Pip/taz 88%, Gentamicin 95%, Cefuroxime 98%, Cefpodoxime 98% and Ceftazidime 100% susceptibility. For patients with 1 risk factor Ciprofloxacin had 80%, Pip/taz 80%, Gentamicin 88%, Cefuroxime 78%, Cefpodoxime 78% and Ceftazidime 83% susceptibility. For 2 or more risk factors Ciprofloxacin drops its susceptibility to 52%, Cefuroxime to 54% and Cefpodoxime to 61%. Pip/taz, Gentamicin and Ceftazidime remain at 75% and 77%, respectively.

**Conclusions:**

We identified several risk factors for resistances and MDR in UTI. Susceptibility towards antimicrobials depends on these risk factors. With no risk factor cephalosporins seem to be the best choice for empiric therapy, but in patients with risk factors the beta-lactam penicillin Piperacillin with Tazobactam is an equal or better choice compared to fluoroquinolones, cephalosporins or gentamicin. This study highlights the importance of monitoring local resistance rates and its risk factors in order to improve empiric therapy in a local environment.

## Background

Urinary tract infections (UTI) are among the most common bacterial infections worldwide. Their therapy is becoming more challenging as resistance rates for standard antibiotics are increasing [1]. The increase of antibiotic resistances and multi-drug resistance (MDR) pathogens in UTI is associated with higher rates of inadequate empirical therapy due to impaired antibiotic coverage [2].

Therefore, early identification of patients at risk for antibiotic resistances is an important aspect for effective treatment. Preceding studies identified a variety of risk factors mainly for treatment failure for either fluoroquinolones or trimethoprim-sulfamethaxol or for UTI with MDR pathogens. Rarely did they quantify the impact of risk factors on overall susceptibility to standard empirical therapy choices [3–8].

The goal of our study was to identify risk factors associated with several antibiotic resistances and MDR pathogens in patients presenting with UTI, mainly pyelonephritis or urosepsis, to the emergency department (ED) of a German academic tertiary care facility. We hypothesized that the identification of risk factors will improve empiric antibiotic therapy in the ED.

## Methods

The study was retrospectively conducted with anonymized patient data from the ED of an academic tertiary care facility. The local ethics committee of the Medical Faculty of Mannheim has approved the study. Cases were eligible for the study, if they were diagnosed with lower or upper UTI in the ED between January 2013 and June 2015. Lower UTI was defined as dysuria, pollakisuria or positive leucocyte and nitrite in urine in patients with reduced vigilance. Upper UTI was defined by additional flank pain, fever, positive systemic inflammation serum parameters or perinephritic abscess in sonography [9].

Patient information included the following: demographic parameters (gender, age, residence), laboratory analysis (C-reactive protein, leucocyte count, serum creatinine and glomerular filtration rate (GFR) calculated after Modification of Diet in Renal Disease formula), physical condition at admission (signs of exsiccosis, symptoms at presentation), urine analysis (isolated pathogen, pathogen count, antimicrobial susceptibility testing), comorbidities (diabetes mellitus, indwelling urinary catheter, renal transplantation, dialysis), pre-ED antibiotic treatment within the last 30 days, prior hospitalization within the last 30 days and UTI within the last 12 months.

We assessed patients with clinical symptoms consistent with UTI and positive urine culture. A positive result was defined as colony-forming unit > 10^4^/ml for catheter- or midstream-urine and > 10^3^/ml for single-use-catheter. Urine cultures labeled by the microbiology laboratory as “contamination” or “mixed flora” were excluded.

MDR and extensively drug-resistant (XDR) pathogens were defined according to the European Centre for Disease Prevention and Control [10]: MDR describes pathogens non-susceptible to at least one agent in three or more antimicrobial categories. XDR describes pathogens fully susceptibly to only two or less antimicrobial categories.

Data were grouped by presence of antibiotic resistances towards antimicrobial substances frequently used in the treatment of UTIs. We chose Ciprofloxacin (Cip), Ceftazidime, Cefpodoxime, Gentamicin, Piperacillin with Tazobactam (Pip/taz) and Cefuroxime for further analysis. Risk factors for carbapenem non-susceptibility were not analyzed as overall non-susceptibility was low. Differences in parameters between groups with or without resistances towards a certain antibiotic were tested for significance with Fisher’s exact testing.

Logistic regression was then performed to identify risk factors for resistances toward antimicrobial substances mainly used in upper UTI treatment, MDR pathogens and pathogens simultaneously resistant to Ciprofloxacin, Pip/taz and Ceftazidime (sCPC). The results were presented with odds ratios and their corresponding 95% confidence interval (CI). The area under the curve (AUC)/c-statistic of a Receiver Operating Characteristic (ROC) analysis was calculated and used to estimate the accuracy of fit of our model. The AUC measures the accuracy for the prediction with an AUC = 1.0 representing perfect prediction.

Susceptibility data were calculated for Ciprofloxacin, Ceftazidime, Cefpodoxime, Cefuroxime, Pip/taz, Gentamicin and Imipenem. Susceptibility was analyzed for the whole patient collective as well as subgroups defined by the number of risk factors present.

All analyses were performed using SAS system for Microsoft version 9.4. (SAS Institute Inc., Cary, North Carolina, USA).

## Results

### Demographics and pathogen distribution

Between January 2013 and June 2015, the ED treated and admitted 469 cases, who met the criteria of UTI. In 184 cases no urine culture was performed, 143 urine cultures had a negative result or were contaminated. In 5 cases the initial diagnosis of upper UTI could not be confirmed during hospital stay. One hundred thirty-seven had an uncontaminated urine culture with a positive result, of which 130 (94.9%) met the criteria for upper UTI. These patients were mostly female (80/137, 58.4%) with a median age of 76.0y (average 72.1y, range 17-97y). Twenty-seven patients resided in nursing homes (19.7%), 22 had indwelling urinary catheters (16.1%), 33 were admitted to a hospital within the last 30 days (24.1%) and 20 received antibiotic treatment within 30 days prior to their ED visit (14.6%; Table 1). In all patients, symptoms of infection started before admission, thus presenting either community acquired UTI or community-onset healthcare-associated urinary tract infections.

**Table 1:** Demographic parameters of patients with a positive urine culture

	All (*n* = 137)	
	*n*	Percentage
Age		
Mean	72.1	(18.2)
< 65 years	30	(21.9)
65–80 years	55	(40.1)
> 80 years	52	(38.0)
Gender		
female	80	(58.4)
Nursing home		
yes	27	(19.7)
Hospitalization within 30 days		
yes	33	(24.1)
Antibiotic treatment within 30 days		
yes	20	(14.6)
Recent UTI		
yes	30	(21.9)
Diabetes		
yes	47	(34.3)
Renal transplantation		
yes	4	(2.9)
Indwelling urinary catheter		
yes	22	(16.1)
Hemodialysis		
yes	4	(2.9)
Exsiccosis		
yes	40	(29.2)
Fever		
yes	50	(36.5)
leucocyte count		
> 12 /nl	56	(40.9)
GFR (from serum creatinine)		
< 15 ml/min	5	(3.7)
< 60 ml/min	79	(57.7)
C-reactive protein		
> 100 mg/l	64	(46.7)

*GFR* glomerular filtration rate

Of 137 urine cultures, main pathogens were *Escherichia coli* in 64.2% (*n* = 88), *Klebsiella pneumoniae* or *oxytoca* in 12.4% (*n* = 17), *Enterococci spp.* in 5.1% (*n* = 7), *Pseudomonas aeruginosa* in 5.1% (n = 7), *Proteus mirabilis* in 4.4% (*n* = 6), *Staphylococcus aureus* in 3.7% (*n* = 5) and *Citrobacter spp.* in 2.2% (*n* = 3) of cases.

All samples were tested for susceptibility towards Ciprofloxacin (Cip), Piperacillin with Tazobactam (Pip/taz), Gentamicin, Cefuroxime, Cefpodoxime and Ceftazidime and Imipenem. In our study 36.5% (50/137) of pathogens can be classified as MDR. Most MDR pathogens were *E. coli* (62.7%) followed by *K. pneumonia* (13.7%). XDR pathogens were detected in 3.7% (5/137). Non-susceptibility to all three antibiotics Ciprofloxacin, Pip/taz and Ceftazidime (sCPC), which are commonly used for the empiric therapy of severe pyelonephritis, was detected in 5.1% (7/137).

### Risk factors for antibiotic non-susceptibility

An analysis of patient characteristics revealed residence in nursing homes, prior hospitalization within 30 days, usage of antibiotics 30 days prior to admission, indwelling urinary catheter, recent or recurrent UTI, gender, renal transplantation and serum leucocyte count > 12.0/nl as significantly different between patients with and without non-susceptibility (*p* < 0.05 in Fisher’s exact testing; data not shown). Logistic regression was performed to allocate risk factors to non-susceptibility to a certain antibiotic, MDR pathogen or to simultaneous non-susceptibility towards Ciprofloxacin, Pip/taz and Ceftazidime (sCPC). Seven factors showed a significant association (Table 2). ROC analysis yielded AUC values of 0.650 to 0.868 indicating an overall good fit of our models.

**Table 2:** Logistic regression modelling presented as Odds Ratio with 95% confidence interval and corresponding AUC

Target	Nursing home residence	Hospitalization within 30 days	Male sex	Renal transplantation	Indwelling urinary catheter	Use of antibiotics within 30 days	Recurrent UTI	AUC
Pip/taz	n.s.	3.7 (1.4–9.5)**	n.s.	15.4 (1.4–172.1)*	n.s.	n.s.	n.s.	0.699
Ciprofloxacin	n.s.	4.4 (1.8–10.6)**	n.s.	n.s.	5.2 (1.8–14.7)**	n.s.	n.s.	0.749
Gentamicin	n.s.	n.s.	n.s.	24.8 (2.4–257.2)**	3.1 (1.0–9.4)*	n.s.	n.s.	0.650
Cefuroxime	n.s.	n.s.	7.3 (2.9–18.5)***	n.s.	n.s.	5.7 (1.8–17.7)**	n.s.	0.792
Cefpodoxime	n.s.	n.s.	6.5 (2.5–17.0)***	n.s.	n.s.	5.3 (1.7–16.3)**	n.s.	0.788
Ceftazidime	n.s.	n.s.	3.7 (1.3–10.6)*	16.4 (1..5–182.1)*	n.s.	n.s.	n.s.	0.715
MDR	n.s.	3.6 (1.5–8.5)**	n.s.	n.s.	n.s.	n.s.	4.0 (1.7–9.8)**	0.707
sCPC	22.8 (3.4–151.2)**	n.s.	9.5 (1.4–62.5)*	n.s.	n.s.	n.s.	n.s.	0.868

* *p* < 0,05; ** *P* < 0,01; ***p < 0,001; *n.s*. not significant, *UTI* urinary tract infection, *AUC* Area under the curve, *Pip/taz* Piperacillin/Tazobactam, *MDR* multidrug resistance, *sCPC* simultaneous non-susceptibility for Pip/taz, Ciprofloxacin and Ceftazidime

### Antimicrobial susceptibility in relation to risk factors

Overall susceptibility for commonly used antibiotics in upper UTI was low with 71.5% for Cip, 80.3% for Pip/taz, 84.6% for Gentamicin, 73.7% for Cefuroxime, 76.6% for Cefpodoxime and 85.4% for Ceftazidime (Table 3). Ampicillin with Sulbactam had an overall susceptibility of 54.7% and was therefore not further analyzed. Only Imipenem showed an acceptable susceptibility rate of 96.4%.

**Table 3:** Susceptibility in % in relationship to number of risk factors (RF)

	Overall	0 RF	1 RF	> = 2 RF
	(*n* = 137)	(*n* = 41)	(*n* = 40)	(*n* = 56)
Ciprofloxacin	71.5%	90.2%	80.0%	51.8%
Pip/taz	80.3%	87.8%	80.0%	75.0%
Gentamicin	84.7%	95.1%	87.5%	75.0%
Ceftazidime	85.4%	100%	82.5%	76.8%
Cefpodoxime	76.6%	97.6%	77.5%	60.7%
Cefuroxime	73.7%	97.6%	77.5%	53.7%
Imipenem	96.4%	100%	100%	91.1%

**Fig. 1:**
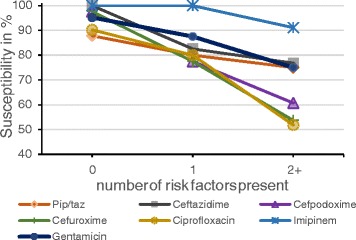
Susceptibility in relationship to number of risk factors - Susceptibility in % for Piperacillin/Tazobactam (red), Ceftazidime (grey), Cefpodoxime (violet), Cefuroxime (green), Ciprofloxacin (yellow), Imipinem (light blue) and Gentamicin (dark blue) are plotted for 0, 1 and 2 and more (2+) risk factors present

In order to recommend non-carbapenem antibiotics for the treatment of upper UTI in the ED we analyzed susceptibility rates by previously identified risk factors. Without a risk factor present all antibiotics had a susceptibility rate of around 90% and above (*n* = 41). If one of seven risk factors were present, susceptibility for all antibiotics except for Imipenem dropped to around 80% (*n* = 40) (Fig. 1). For 2 or more risk factors Cip, Cefuroxime and Cefpodoxime dropped to 60% and below, while Pip/taz, Gentamicin and Ceftazidime remained at around 75% (*n* = 56). In this case, the susceptibility rate of Imipenem also dropped to 91.1% (Table 3). Whereas cephalosporins and Gentamicin seem to be preferable in patients with no risk factors in regard to susceptibility, their susceptibility rates were similar to Pip/taz and Cip in patients with one risk factor. Interestingly, Pip/taz, Gentamicin and Ceftazidime kept their susceptibility rate relatively stable with more risk factors whereas Cip, Cefuroxime and Cefpodoxime dropped their susceptibility rates to a greater extent.

The logistic regression modelling showed that not all antibiotics share the same risk factor profile (Table 2), therefore sub analysis was performed. In our population, patients, who have been admitted to a hospital within the previous 30 days and resided in a nursing home (*n* = 9), showed a much better susceptibility rate for cephalosporins (77.8%) compared to Gentamicin (55.6%), Pip/taz (33%) or Ciprofloxacin (11%). In contrast, patients with antibiotic therapy 30 days prior to admission (*n* = 20) had a high susceptibility rate for Pip/taz 95% compared to Gentamicin (80.0%), Cefuroxime 45.0%, Cefpodoxime 50.0%, Ceftazidime 75.0% and Ciprofloxacin 60.0%. Our data suggest that patients with previous antibiotic therapy may be better treated with Pip/taz as empiric therapy.

### Therapy algorithm

**Fig. 2:**
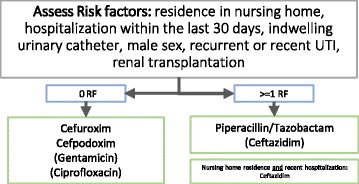
Therapy algorithm for our emergency department - First-line therapy based on susceptibility rates for patients with zero risk factors (left) and patients with one or more risk factors (right)

From our data we concluded for our ED that for patients without a risk factor a cephalosporin-based empiric treatment or Gentamicin has the lowest rate of non-susceptibility (Fig. 2). However, with one or more risk factors Pip/taz is equal to or better as cephalosporins or Ciprofloxacin, particularly in case of prior antibiotic therapy. An exception could be patients residing in a nursing home and who have been admitted to a hospital in the previous 30 days. They could benefit from Cefpodoxime or Cefuroxime as an initial treatment choice.

In our study, only 80 of 124 patients (64.5%) received an empiric therapy that showed susceptibility to its causing pathogen in urine culture testing. Thirty-three patients received a cephalosporin as empiric therapy, 10 Pip/taz, 15 Ciprofloxacin, but 54 Ampicillin/Sulbactam. An empiric therapy based on common guidelines for upper UTI such as ciprofloxacin would have an overall susceptibility of 71, 5% (Table 3). Following our risk factor based decision algorithm, 86.1% (107/124) would have received a susceptible antibiotic treatment. In this algorithm, patients with one or more risk factors would have been treated with Pip/taz except for patients, who had been admitted to the hospital within the last 30 days prior to admission and reside in a nursing home, which would have received a cephalosporine. All patients without a risk factor would have received Cefpodoxime or Cefuroxime as first line therapy (Fig. 2).

## Discussion

With the increase of microbial resistance, empiric therapy recommendations without taking local resistance data into account can lead to inferior treatment results. In our data set treating patients with upper UTI per current treatment guidelines would have led to incorrect antibiotic coverage in nearly 30% of cases [9]. Early identification of patients at risk of antibiotic resistances and thus therapy failure is an important part of an effective empiric therapy.

In the past risk factors for extended-spectrum-betalactamase producing bacteria in non-hospitalized patients with UTI have for example been identified as recent hospitalizations within 3 months, previous antibiotic usage, age > 60 years, diabetes mellitus, male gender, previous UTI with *Klebsiella spp.*, residence in long-term care facilities, indwelling urinary catheters, recurrent UTIs and previous fluoroquinolone use [11, 12]. Analogous results were obtained for fluoroquinolones [3, 6–8, 13]. In a similar setting to ours, Faine et al. evaluated risk factors for MDR pathogens in UTI in the United States [14]. Their overall MDR rate was only 6.7% in contrast to 36.5% in our population. Faine et al. used a more restrictive MDR definition, which may explains the observed difference in MDR rates and they identified male gender, chronic hemodialysis and nursing home residence as risk factors [14].

We identified several risk factors for UTI with non-susceptible pathogens in our study. They are similar to those previous identified risk factors such as prior hospitalization within 30 days, residence in nursing homes, recurrent UTI, male gender, renal transplantation, permanently indwelling urinary catheter and prior usage of antibiotics within the last 30 days. Our analysis considered several antibiotics and demonstrates that not all risk factors are associated with non-susceptibility to all antimicrobial categories. For example, residing in a nursing home was the only significant risk factor for infection with MDR pathogens, whereas hospitalization within 30 days and indwelling urinary catheter were significant for Ciprofloxacin resistance. This seems consistent with published data that showed various risk factors associated with different antimicrobial categories in different studies [3, 13, 15]. A large multicenter, prospective study would be necessary to further explore, which risk factors cause non-susceptibility to certain antimicrobial categories.

Stratification of empiric antibiotic therapy by risk factors can improve pathogen coverage significantly. In our study, susceptibility to standard antibiotics was overall less than 85%. However, when analyzed by presence of risk factors our data showed that patients with no risk factors had > 85% susceptibility for all standard antibiotics. Patients with one risk factor present had still a susceptibility rate of 77.5 to 82.5% for cephalosporins and 80.0% for Pip/taz. Patients with two or more risk factors remained only susceptible to Imipenem, but its susceptibility for Pip/taz, Gentamicin and Ceftazidime was reasonable with 75.0% or 76.8%, respectively.

Interestingly, in our study antibiotic use within the last 30 days was associated with a higher resistance rate in cephalosporins and Ciprofloxacin compared to the beta-lactam penicillin Pip/taz consisted with observations that cephalosporins and fluoroquinolones may increase the resistance rates of bacteria [16]. Our data suggest that patients with previous antibiotic therapy may be better treated with Pip/taz empirically although the number of patients in this sub-analysis was low in order to draw a statistical sound conclusion.

There are several important limitations to consider in our study. It was a single-center, retrospective data analysis, which depended on the accuracy of history taking by the health care provider on call. Certain published risk factors, such as employment in health care, exposure to farming, family members with multidrug resistant pathogens, ambulant chemotherapy and wound care, had to be excluded from our study as they have not been recorded consistently in all cases. A selection bias towards antibiotic resistance cannot be excluded as not all patients presenting with UTI received a urine culture or had a positive urine culture result. Overall, the rate of antibiotic resistance or MDR pathogens was high compared to similar studies.

However, our results are among the first to show risk factors for antibiotic resistances and MDR pathogens in UTI patients in Germany. In contrast to most studies, we analyzed patients admitted to an emergency department in a large tertiary care hospital and did not solely focus on a single antibiotic substance or on MDR pathogens. Our results show that implementation of risk factors can lead to significant improvement in susceptibility in empirical therapy.

Our study revealed also another important aspect as patients diagnosed with upper UTI in the ED were admitted to the ward with an empiric antibiotic therapy, but the sampling of urine cultures was often referred to the ward nursing team due to logistical reasons. This led to a high number of negative urine cultures as the antibiotic treatment was applied before urine cultures were taken. Studies like these can help not only to identify local resistant patterns or risk factors for resistant bacteria, it also helped us to identify serious organizational problems in the daily routine.

Current treatment guidelines often recommend fluoroquinolones and cephalosporins as treatment options in uncomplicated upper UTI. Both antimicrobial categories should be viewed critical, because they lead to a significant increase of *Clostridium difficile* colitis and may further increase the rate to MDR pathogens [16, 17]. Piperacillin/Tazobactam is less prone to induce antibiotic resistance [18]. It also inhibits *C. difficile* colonization during therapy [19]. In this regard, Pip/Taz is the better antibiotic choice. Gentamicin can cause severe side effects, such as kidney and inner ear damage and needs extended monitoring in comparison to other antibiotics especially in elderly patients. Therefore, in our institution we do not recommend Gentamicin for empiric therapy in upper UTI infections.

β-Lactamase inhibitors (BLIs) such as Tazobactam play an important role in overcoming β-lactam resistance in Gram-negative bacteria. Because of the emergence of varieties of β-lactamases, their effectiveness has diminished over time. New BLI combinations with broad-spectrum antibiotics are promising for increasing the effectiveness of empiric antibiotic therapy in UTI [20]. Ceftolozane/tazobactam and ceftazidime/avibactam have been approved for UTI treatment and increase the overall susceptibility to gram-negative bacteria. In particular, ceftazidime/avibactam has been shown to be effective in isolates from UTI patients resistant to Pip/Taz, Cephalosporins and Carbapenems [21, 22]. In our study, bacteria were not routinely tested for these new ß-lactamase inhibitor combinations as they are regarded as last line therapies in severely ill patients due to highly resistant bacteria.

In our setting, all standard antibiotics had a relatively low overall susceptibility. A simple risk factor based treatment algorithm using cephalosporins in patients without risk factors and Pip/taz in all other patients except for patients residing in nursing homes with recent hospitalization in the previous 30 days increases the antibiotic coverage rate to 86.1%. It can be debated whether the use of Pip/taz in all cases as a broad-spectrum beta-lactam penicillin derivative with an overall susceptibility rate of 80.3% would be much different in its clinical outcome. With its advantages regarding *C. difficile* colonization and induction of resistances Pip/taz could be the better choice compared to cephalosporins and fluoroquinolones for urinary tract infections.

## Conclusions

We retrospectively identified seven independent risk factors for antimicrobial resistances in UTI patients in the local emergency department: prior hospitalization within 30 days, residence in nursing homes, recurrent UTI, male gender, renal transplantation, permanently indwelling urinary catheter and prior usage of antibiotics within the last 30 days. Antibiotic susceptibility changes significantly in regard to risk factors present in a patient population. These results can be used to improve empirical antibiotic therapy in emergency departments with high rates of resistances. The beta-lactam penicillin-derivate piperacillin/tazobactam is likely the better choice compared to fluoroquinolones and cephalosporins as susceptibility in patients with risk factors is comparable or better. Whether the incidence of *C. difficile* colitis and the increase of multi-resistant bacteria can be reduced by treatment with piperacillin/tazobactam should be explored in further randomized, prospective multi-center studies. We encourage readers to monitor their local susceptibility rates to choose appropriate empirical therapy, as the local situation may significantly differ from guideline and literature results.

## Abbreviations

AUC: Area under the curve
BLI: β-Lactamase inhibitor
Cip: Ciprofloxacin
ED: Emergency department
GFR: Glomerular filtration rate
MDR: Multidrug-resistant
Pip/taz: Piperacillin/tazobactam
sCPC: Simultaneous resistance towards Ciprofloxacin, Piperacillin/tazobactam and Ceftazidime
UTI: Urinary tract infection
XDR: Extensively drug-resistant
